# Metabolic regulation of oocyte maturation and early embryo development in mammals

**DOI:** 10.1093/lifemeta/loag024

**Published:** 2026-08-06

**Authors:** Sifan Rong, Yixuan Fu, Yan Li, Yingxin Celia Jiang, Lanruo Chen, Yue Zhao, Wencheng Zhu, Liangshan Mu

**Affiliations:** Reproductive Medicine Center, The First Affiliated Hospital of Wenzhou Medical University, The First School of Medicine, Wenzhou Medical University, Wenzhou, Zhejiang 325000, China; Reproductive Medicine Center, The First Affiliated Hospital of Wenzhou Medical University, The First School of Medicine, Wenzhou Medical University, Wenzhou, Zhejiang 325000, China; Reproductive Medicine Center, The First Affiliated Hospital of Wenzhou Medical University, The First School of Medicine, Wenzhou Medical University, Wenzhou, Zhejiang 325000, China; Department of Reproductive Medicine, Shanghai Key Laboratory for Assisted Reproduction and Reproductive Genetics, Renji Hospital, Shanghai Jiao Tong University School of Medicine, Shanghai 200127, China; Institute of Molecular Physiology, Shenzhen Bay Laboratory, Shenzhen, Guangdong 518132, China; State Key Laboratory of Female Fertility Promotion, Center for Reproductive Medicine, Department of Obstetrics and Gynecology, Peking University Third Hospital, Beijing 100191, China; National Clinical Research Center for Obstetrics and Gynecology (Peking University Third Hospital), Beijing 100191, China; Key Laboratory of Assisted Reproduction, Ministry of Education, Beijing 100191, China; Beijing Key Laboratory of Collaborative Innovation in Frontier Technologies for Population Quality, Beijing 100191, China; Institute of Molecular Physiology, Shenzhen Bay Laboratory, Shenzhen, Guangdong 518132, China; Reproductive Medicine Center, Zhongshan Hospital, Fudan University, Shanghai 200032, China

**Keywords:** embryo metabolism, oocyte metabolism, metabolic regulation

## Abstract

Metabolism plays a central role in coordinating mammalian oocyte maturation and early embryonic development. The metabolic changes that occur during these stages are essential for the acquisition of developmental competence and successful reproduction. This review summarizes metabolic regulation from oocyte growth and maturation through fertilization and preimplantation development, with particular emphasis on glucose, lipid, and amino acid metabolism, as well as mitochondrial function. We also discuss how metabolic disturbances associated with maternal obesity, ovarian aging, and polyendocrine metabolic ovarian syndrome (formerly termed polycystic ovary syndrome) impair oocyte quality and embryonic developmental potential. By integrating recent insights from multi-omics, live-cell imaging, and genetic studies, we propose a framework for understanding how metabolism not only supports but also actively governs developmental decisions. We further discuss mechanism-based strategies to restore metabolic balance and improve reproductive outcomes.

## Introduction

Metabolism is far more than a source of energy. It also dynamically regulates cell fate, signaling, and developmental potential. In mammalian reproduction, precise metabolic control underlies oocyte maturation, fertilization, and preimplantation development. Metabolites serve as signaling molecules, epigenetic cofactors, and biosynthetic precursors that shape developmental timing and competence. Oocyte maturation and early embryonic development represent critical stages in the beginning of life, and investigating their metabolic regulatory mechanisms is of great significance for uncovering the molecular basis of clinical issues such as reproductive aging and infertility. Oocyte maturation and early embryogenesis involve a series of highly regulated events, including meiotic resumption, fertilization-induced oocyte activation, and embryonic lineage specification before implantation. Each of these stages is associated with distinct energetic requirements and extensive metabolic remodeling. For example, preimplantation embryos exhibit stage-specific substrate preferences: while zygotes primarily rely on pyruvate and oxidative phosphorylation, the blastocyst stage shifts toward glucose-driven aerobic glycolysis (the Warburg effect) [1–3]. This metabolic transition is closely linked to mitochondrial dyna­mics, reactive oxygen species (ROS) homeostasis, and epigenetic reprogramming. In terms of metabolic pathways, glucose, lipids, and amino acids are intricately connected through the tricarbo­xylic acid (TCA) cycle. During oocyte maturation, mitochondrial TCA cycle activity is markedly upregulated during meiotic resumption, generating nicotinamide adenine dinucleotide (NADH) and flavin adenine dinucleotide (FADH_2_) to supply ATP for chromosome segregation [2]. Lipid droplets (LDs), as key energy reservoirs, contribute to energy metabolism via β-oxidation of fatty acids, producing acetyl coenzyme A (acetyl-CoA) that not only fuels energy production but also modulates chromatin structure through histone acetylation [4]. Recent technological advances in low-input or single-cell metabolomics, combined with live-cell imaging, have enabled the construction of dynamic metabolic maps covering the entire preimplantation period in mouse embryos (Table 1). Integration with epigenomic, transcriptomic, and newly delineated single‑embryo proteomic data has further revealed the metabolic regulatory mechanisms underlying zygotic genome activation (ZGA) in mice [3, 5, 6]. Additionally, newly established lipidomic maps capturing the dynamic changes in lipid profiles during early embryonic development in both mice and humans have elucidated the functional roles and mechanisms of lipid unsaturation in regulating embryogenesis [7]. These metabolic maps contribute to a better understanding of the dynamic regulation of metabolism during this critical period.

**Table 1:** Recent metabolomic profiling studies of oocyte maturation and preimplantation embryo development.

References	Species	Developmental stage and sample size	Detected number of metabolites	Method
Li *et al.* [10]	Mouse	GV, GVBD, and MII *n *= 2000 per replicate, 9 replicates per stage	Unspecified	UHPLC-HRMS
Chi *et al.* [14]	Mouse	Morula *n *= 250 per replicate, 3 replicates	Approximately 100 metabolites	(i) HPLC-MS (ii) ICS-MS
Sharpley *et al.* [2]	Mouse	2C, morula, and blastocyst *n *> 250 per replicate, 3 replicates per stage	Unspecified	(i) Stable isotope resolved metabolomics (ii) LC-MS
Zhao *et al.* [31]	Mouse	2C and blastocyst *n *= 100 per replicate, 3 replicates per stage	256 metabolites	UHPLC-MS
Li *et al.* [5]	Mouse	Zygote, 2C, 4C, 8C, morula, and blastocyst *n *= 1000 per replicate, 6 replicates per stage	246 metabolites	UHPLC-Q-TOF-MS
Zhang *et al.* [7]	Mouse and Human	Mouse: MII oocyte, zygote, 2C, 4C, 8C, and blastocyst *n *= 120 per replicate MII to 2C, 4 replicates per stage 4C to blastocyst, 3 replicates per stage Human: 8C and blastocyst n = 30 per replicate, 4 replicates per stage Human: 8C and blastocyst *n *= 30 per replicate, 4 replicates per stage	332 metabolites	UPLC-MS/MS

2C = 2-cell stage; 4C = 4-cell stage; 8C = 8-cell stage.

**Figure 1:**
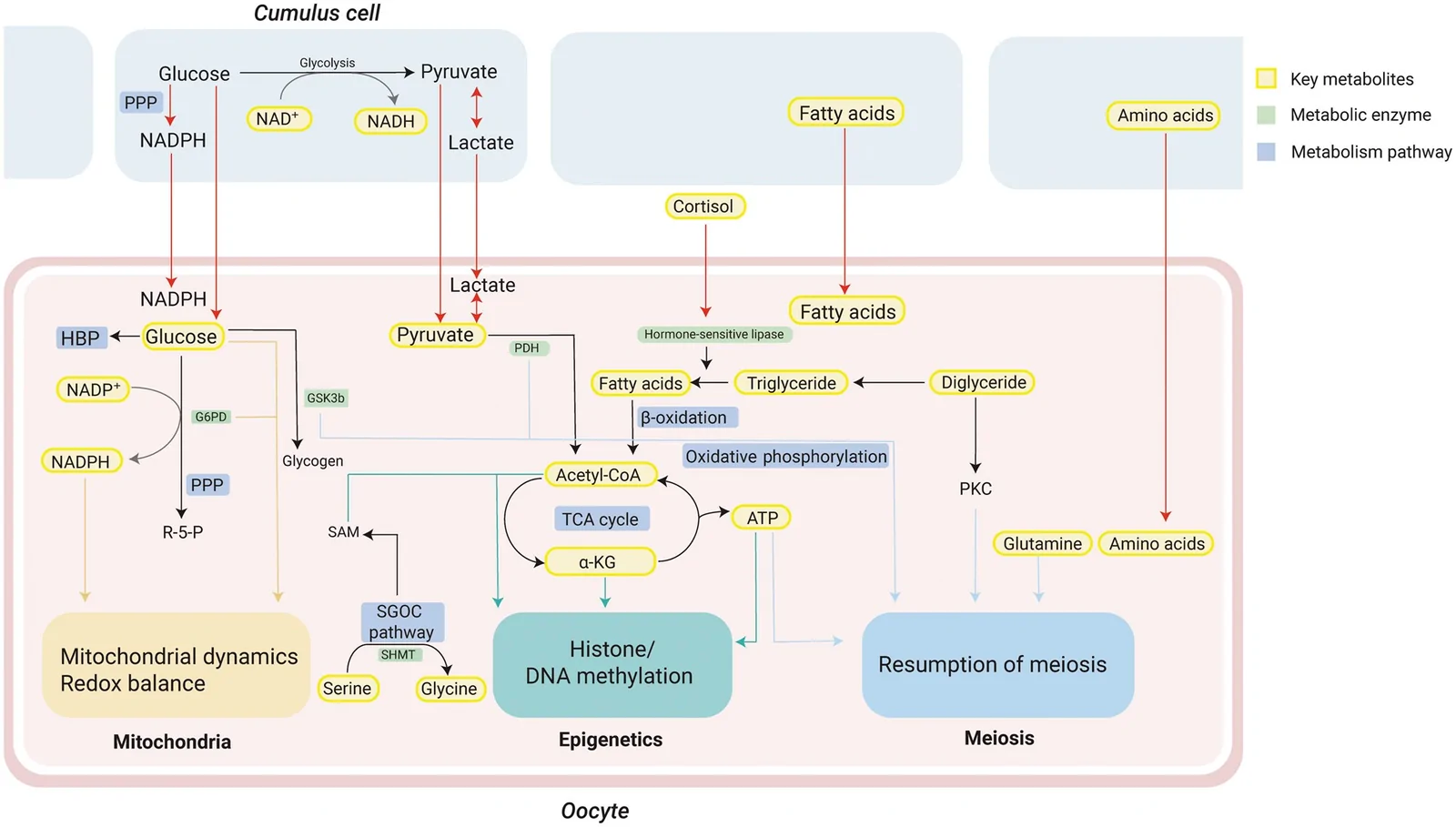
Metabolic and epigenetic regulation during oocyte maturation. Schematic illustration of the key metabolic pathways and metabolite fluxes that regulate oocyte maturation is shown. Metabolites such as glucose, pyruvate, amino acids, cortisol, and lipids are exchanged between cumulus cells and the oocyte.

Beyond intrinsic metabolic reprogramming within the oocyte, metabolic symbiosis with surrounding cumulus cells is a criti­cal determinant of oocyte competence. Unlike somatic cells, oocytes possess limited glycolytic capacity and rely heavily on the cumulus-oocyte complexes (COC) for the uptake of essential metabolites. Specifically, cumulus cells actively metabolize glucose to generate pyruvate and lactate, which serve as the primary oxidative substrates for the oocyte. Concurrently, the pentose phosphate pathway (PPP) in cumulus cells produces NADPH, which is transferred to the oocyte to maintain redox homeostasis. Furthermore, cumulus cells supply fatty acids and amino acids, fueling β-oxidation and one-carbon metabolism, respectively. This metabolic coupling ensures that the oocyte conserves its resources for biosynthesis and epigenetic modifications while delegating the bulk of catabolic processes to the supportive granulosa-derived cumulus cells (Fig. 1).

This review focuses on the mechanisms of metabolic reprogramming from oogenesis to early embryogenesis, with particular attention to changes in glucose metabolism, lipid metabolism, amino acid metabolism, and mitochondrial function. Moreover, we propose a framework in which developing oocytes and embryos do not merely consume nutrients but also actively utilize dynamic changes in metabolites as a form of “metabolic language.” This language orchestrates developmental competence by influencing epigenetic states and signaling pathways. We also explore the molecular links between metabolic dysregulation and reproductive disorders such as obesity, ovarian aging, and polyendocrine metabolic ovarian syndrome (PMOS), offering new insights and directions for future research.

## Glucose metabolism as a dynamic driver of oocyte competence and embryonic cell fate

**Figure 2:**
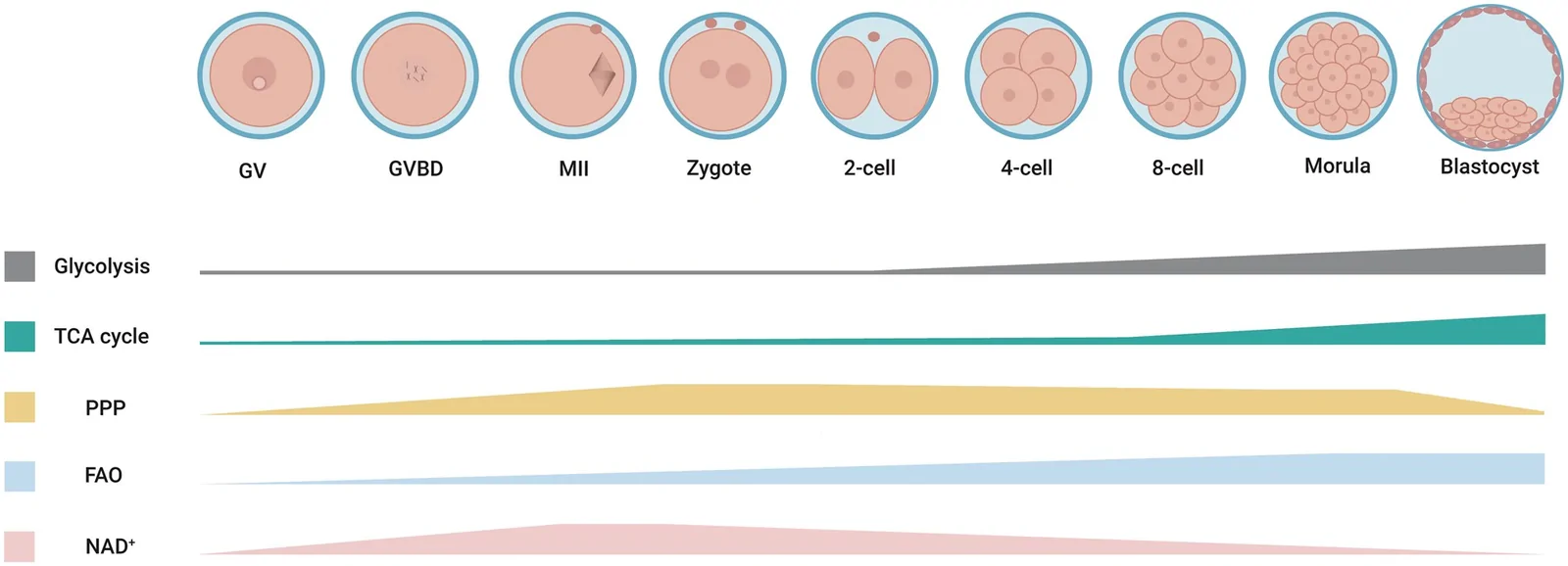
Metabolic profile during oocyte maturation and preimplantation embryonic development. Schematic illustration of mammalian oocytes progressing through key maturation stages followed by fertilization and preimplantation embryonic development is shown. The relative activity of major metabolic pathways across these stages is indicated by the colored gradients.

Glucose metabolism plays a central role in the successful progression from oocyte maturation to blastocyst formation, impacting not only energy production but also key developmental decisions and epigenetic regulation. Although glycolysis is the initial energy source in most cell types, oocytes exhibit strikingly low glycolytic activity, particularly during meiotic maturation [8]. Both glyco­lytic enzymes and intermediates remain at low levels during this period, indicating that glycolysis is not the principal energy path­way [8]. To compensate for the limited glycolytic capacity of the oocyte, cumulus cells within the COCs metabolize glucose through glycolysis and supply the resulting metabolites to the oocyte. This process yields high concentrations of pyruvate and lactate, which diffuse through gap junctions into the oocyte, directly entering the mitochondrial TCA cycle to sustain ATP production during meiotic maturation (Fig. 1). In contrast, the TCA cycle is markedly upregulated during oocyte maturation (Fig. 2). As the central hub of aerobic metabolism, the TCA cycle efficiently generates ATP from pyruvate and provides biosynthetic intermediates [9]. During oocyte maturation, the abundance of several key TCA metabolites—such as citrate, succinate, and fumarate—as well as critical enzymes, including pyruvate dehydrogenase (PDH) subunit 1, dihydrolipoyl transacetylase, and dihydrolipoyl dehydrogenase, is markedly increased [10], with enzymatic activity notably enhanced during meiotic resumption. This metabolic activation not only improves ATP generation efficiency through oxidative phosphorylation but also provides essential acetyl-CoA substrates for epigenetic modifications such as histone acetylation.

The PPP is similarly active during oocyte maturation (Fig. 2). By generating NADPH and ribose-5-phosphate (R-5-P), the PPP supports both redox balance and nucleotide biosynthesis [11]. While the oocyte retains an intrinsic PPP, a significant fraction of NADPH is sourced from the cumulus cells (Fig. 1). Key intermediates, such as 6-phosphogluconate and ribulose-5-phosphate, along with related enzymes such as glucose-6-phosphate dehydrogenase (G6PD), are significantly elevated during this process [10]. Notably, the levels of two major upstream PPP metabolites—glyceraldehyde and sorbitol—decrease during meiosis and increase during meiotic resumption, followed by a reduction at the metaphase II (MII) arrest stage [10]. Meanwhile, PPP activity is closely linked to the redox homeostasis of oocytes. Inhibition of G6PD leads to ROS accumulation and impaired oocyte development, underscoring the protective role of PPP in redox homeostasis [10].

Overall, glucose metabolism undergoes dynamic reprogramming during oocyte maturation: glycolytic capacity is limited, while the TCA cycle and PPP are concurrently activated. The TCA cycle drives ATP synthesis and supplies metabolites for epigenetic reprogramming. Concurrently, the PPP supports nucleotide biosynthesis and redox defense via NADPH generation. This coordinated strategy balances energetic, biosynthetic, and epigenetic demands to prepare the oocyte for maturation. Following fertili­zation, glucose metabolism undergoes further remodeling in the developing embryos (Fig. 2). During preimplantation develop­ment, TCA cycle activity is enhanced at the 8-cell stage and peaks at the blastocyst stage, while glycolysis remains low until the blastocyst forms [5]. Early-stage embryos (from the zygotic stage to the morula stage) display low glucose uptake, limited expression of glucose transporters, and reduced abundance of glycolytic enzymes and intermediates [12]. Instead, pyruvate and lactate serve as the dominant energy substrates, bypassing gly­colysis to fuel the TCA cycle and anabolic processes [10]. Further studies have revealed the pivotal role of TCA cycle metabolites in regulating the first lineage segregation of human embryos. A recent investigation demonstrated that in trophoblast stem cells derived from human embryonic stem cells, intracellular levels of the TCA cycle intermediate α-ketoglutarate (α-KG) are markedly elevated [13]. Intriguingly, this metabolic reprogramming is directly coupled to cell fate determination: exogenous supplementation with α-KG specifically promotes the differentiation of embryonic stem cells toward the trophectoderm (TE) lineage and facilitates the polarization and maturation of blastoids [13]. Mechanistically, this effect is not mediated through the canonical role of α-KG in histone demethylation; rather, it occurs by lowering intracellular acetyl-CoA levels, thereby attenuating histone ace­tyltransferase activity and leading to a global reduction in histone acetylation. Such metabolite-driven epigenetic remodeling weakens the pluripotency network and creates a permissive chromatin environment for the activation of TE-specific genes [13]. This work provides a novel perspective on how metabolites can govern cell fate decisions by directly shaping chromatin states.

Glycolysis becomes increasingly important after blastocyst formation, when it supports ATP production and compensates under pyruvate-limited conditions [14]. Gene expression of glycolytic enzymes is upregulated during this transition, reflecting heightened metabolic flexibility. Moreover, spatially organized glycolytic gradients have been observed in mouse mesoderm [15], suggesting that glycolysis may play instructive roles in patterning, cell differentiation, and lineage commitment—functions that extend beyond bioenergetics. These findings further support the idea that glycolysis is not strictly required during the morula-to-blastocyst transition. However, whether such spatial metabolic patterns exist in human embryos remains to be determined.

The most recent advances have further expanded our understanding of the non-energetic functions of glucose metabolism during embryonic development, revealing its conserved mechanism of exerting an instructive role via metabolism-signaling coupling across multiple key developmental stages [16]. Recent studies employing gastruloid models derived from mouse and human embryonic stem cells have demonstrated that glyco­lytic activity functions not only as an energy source but also as an upstream signal governing germ layer specification [16]. Specifically, inhibition of glycolysis—either by treatment with 2-deoxy-D-glucose (2-DG) or by glucose deprivation—markedly reduces mesoderm and endoderm formation while concomi­tantly favoring ectodermal fate. This regulatory effect is dose-dependent: higher exogenous glucose concentrations promote mesoderm/endoderm specification, whereas lower concentrations bias differentiation toward ectoderm [16]. Mechanistically, glycolysis promotes the expression of *Brachyury* (*T/Bra*) and primitive streak formation by activating Nodal and Wnt signaling pathways, and this instructive role can be uncoupled from its energetic contribution; activation of Nodal or Wnt signaling partially rescues mesoderm and endoderm development even under glycolytic inhibition [16]. This work provides the first clear evidence in mammalian models that a metabolic pathway exerts an “instructive” role in germ layer fate decisions, offering new insights into how glucose concentration can regulate early embryonic patterning through metabolism-signaling coupling.

A recent landmark study has fundamentally reshaped the traditional view of glucose metabolism [14]. During the transition from morula to blastocyst, glucose is not primarily utilized for energy production or lipid/amino acid biosynthesis. Instead, it functions as a signaling molecule through the PPP and the hexo­samine biosynthetic pathway (HBP). HBP-driven O-glycosylation promotes the nuclear localization of Yes-associated protein 1 (YAP1), while PPP-derived nucleotides, together with sphingosine-1-phosphate (S1P) signaling, activate mammalian target of rapamycin (mTOR), thereby enhancing the translation of transcription factor activating protein 2 gamma (TFAP2C). Together, TFAP2C and YAP1 form a transcriptional complex with TEA domain transcription factor 4 (TEAD4) to initiate the expression of TE-specific genes such as caudal-related homeobox transcription factor 2 (*CDX2*), whereas the inner cell mass (ICM) fate remains unaffected by glucose [14]. This mechanism reveals, for the first time, a “non-energetic” role of glucose in the first lineage decision of the embryo, establishing a paradigm for the emerging field of developmental metabolism.

Parallel to these glycolytic dynamics, the PPP contributes prominently during gastrulation in mouse embryos by directing glucose-derived carbon into nucleotide synthesis [2]. In this phase, glucose flux is distributed among the PPP, glycolysis, and the HBP, with recent studies revealing extensive crosstalk among these pathways [17]. The HBP, in particular, has emerged as a critical regulator of cell fate in the epiblast, modulating mesoderm specification and epithelial–mesenchymal transition through glucose sensing and metabolic signaling [17]. As development proceeds, HBP activity declines, and mesodermal cells increasingly rely on glycolysis to support migration and morphogenesis [17]. Although the temporal partitioning between the HBP and glycolysis has been experimentally supported, current findings are largely based on mouse models with limited sample sizes. Whether the HBP plays a similarly dominant role in early fate determination in human embryos, as well as the potential species-specific differences in glycolytic enzyme activity, remains to be elucidated through cross-species studies. Additionally, the interplay between metabolic pathways and epigenetic modifications—such as histone acetylation—may represent a crucial entry point for decoding the coupling between metabolism and cell fate.

Pyruvate and lactate are closely connected during early embryonic development. Pyruvate mainly supports mitochondrial metabolism, whereas lactate can be used as an oxidative substrate after conversion to pyruvate. Recent studies suggest that lactate may also regulate early development beyond its role as an oxidative substrate, including effects on ZGA and cell-fate patterning. Lactate is a key nutrient in the developmen­tal microenvironment, present at high levels in both *in vivo* and *in vitro* conditions and readily oxidized as early as the zygote stage [18]. Recent work has shown that lactate accumulates in the nuclei of early embryos during major ZGA in both humans and mice [19]. Inhibition of lactate production or uptake results in developmental arrest at the 2-cell stage, profound defects in ZGA, and a loss of lactate-derived H3K18 lactylation (H3K18lac) [19]. Mechanistically, H3K18lac is enriched at the promoter regions of most major ZGA genes and strongly correlates with their expression, suggesting that lactate metabolism directly supports transcriptional reprogramming in early embryogenesis [19]. Aligning with this, global post-translational modification profiling has mapped thousands of site‑specific modifications during meiotic maturation that reinforce such metabolic‑epigenetic linkages [6]. A recent study based on embryonic stem cell–derived models has provided further evidence that metabolism exerts an active, instructive role in embryonic fate determination [20]. By integrating single-cell transcriptomics with morphological analyses, the authors demonstrated that the early balance between glycolysis and oxidative phosphorylation dictates the mesoderm–neuroectoderm ratio in trophoblast-like structures. High glycolytic activity promotes mesoderm specification through a lactate–WNT signaling axis, whereas oxidative phosphorylation predominance biases cells toward neuroectodermal fate [20]. Notably, metabolic interventions, such as inhibiting oxidative phosphorylation or supplementing lactate, were sufficient to restore normal morphogenesis in embryonic models, highlighting that metabolic states function not merely as sources of energy but as instructive signals that actively guide embryonic development.

These findings highlight glucose metabolism as a developmentally reprogrammed and context-dependent regulator. During oocyte maturation, glucose utilization is relatively limited and is directed primarily toward biosynthesis and redox homeostasis. After fertilization, glucose metabolism is progressively reconfigured to support energy production and embryonic cell fate specification [8]. Collectively, these findings demonstrate that glucose metabolism plays a central role throughout oocyte and early embryonic development. Its function extends beyond its conventional role in energy provision, evolving into a dynamic and instructive regulatory network. This dynamic plasticity not only sustains developmental progression but also provides a metabolic framework for linking nutrient availability to lineage decisions and epigenetic state.

## Lipid metabolism: from energy provision to developmental patterning

Lipid metabolism plays a pivotal and evolving role across oocyte maturation and early embryonic development, transitioning from a primary energy source during oogenesis to a more specialized role in morphogenesis and spatial patterning during embryogenesis.

During oocyte maturation, lipids serve not only as critical energy substrates but also as modulators of developmental competence. Fatty acids derived from *de novo* synthesis in cumulus cells are transferred to the oocyte (Fig. 1). In general, lipid metabolites are significantly reduced upon meiotic resumption, particularly from the germinal vesicle (GV) to MII stage [10]. Notably, members of the carnitine family—specifically free carnitine and palmitoylcarnitine, which function as key carriers in fatty acid oxidation (FAO)—display a specific increase during meiotic resumption [10]. It is known that mitochondrial FAO is an important component of oocyte energy metabolism and a conserved feature in mammals [21]. Studies have shown that the levels of carnitine and palmitoylcarnitine increase by 3- to 4-fold during meiotic resumption, accompanied by enhanced FAO activity. This is further substantiated by the upregulation of carnitine palmitoyltransferase II (CPTII), a key enzyme mediating mitochondrial fatty acid import for β-oxidation, as well as the increased expression of various enzymes involved in fatty acid degradation in ovulated MII-stage oocytes [10].

Cholesterol metabolism also exhibits distinct changes during oocyte maturation. The levels of cholesterol and bile acids, including glycocholic acid and taurocholic acid, progressively decrease during meiosis [10]. This regulation is biologically significant, as excessive cholesterol has been shown to induce spontaneous oocyte activation and premature exit from MII arrest, compromising fertilization potential [10]. Similarly, polyunsaturated fatty acids (PUFAs)—such as arachidonic acid (ARA), docosahexaenoic acid, and eicosapentaenoic acid—decline during meiotic resumption [10]. These PUFAs, likely released from phospholipids by phospholipase A2 (PLA2), are key substrates for prostaglandin and leukotriene synthesis. The decrease in ARA, in particular, appears necessary to relieve repression of meiotic progression imposed by high ARA, as high ARA levels interfere with chromosome alignment and trigger aneuploidy [10]. Moreover, reduced ARA supports maternal RNA degradation via B cell translocation gene 4 (BTG4) accumulation—an essential step for oocyte maturation [10].

In early embryos, lipid metabolism was traditionally viewed as a passive energy reserve, with preimplantation development thought to rely more on glycolysis and oxidative phosphorylation [22]. However, high-resolution lipidomic analyses have revised this view, revealing complex, stage-specific remodeling of lipid composition. A conserved increase in phospholipid unsaturation is observed from the 2-cell stage to the blastocyst stage, likely facilitating plasma membrane fluidity required for blastocoel formation and cell polarity [7]. LDs, previously considered inert stores, undergo dynamic reorganization. The LD-associated protein cell death-inducing DNA fragmentation factor-like effector A (CIDEA) plays a central role at the blastocyst stage by promoting LD fusion and preventing lipolysis, thus supporting epiblast cavity formation [23].

FAO continues to be metabolically relevant in embryos, particularly under stress or during specialized states. For instance, in embryonic diapause, lipid stores become the dominant energy source [24]. Diapausing embryos utilize FAO for survival, with the transcription factor forkhead box protein O1 (FOXO1) regulating this pathway via L-carnitine metabolism [25]. Exogenous carnitine enhances FAO efficiency and prolongs survival in dormant embryos. Furthermore, lipid desaturation enzymes such as stearoyl-CoA desaturase 1 regulate monounsaturated fatty acid levels (e.g., oleic acid) and influence polarity establishment from the 8-cell stage onward [7].

Recent discoveries also link lipid metabolism to morphogenetic events. For example, under mTOR inhibition, L-carnitine accele­rates rosette-like structure formation, indicating a direct interaction between lipid mobilization and morphogenesis [25]. These findings suggest that beyond energy supply, lipid metabolism directly contributes to cell fate specification, tissue organization, and the developmental plasticity of embryos.

These recent advances reveal a continuous but shifting role of lipid metabolism: from orchestrating energetic and epigenetic readiness in the oocyte to enabling structural and spatial coordination in the embryo. This temporal and functional transformation underscores lipids as multifaceted regulators of reproductive success.

## Amino acid metabolism: coordinating redox state, epigenetics, and energy across the oocyte-to-embryo transition

Amino acid metabolism plays multifaceted and dynamically regu­lated roles throughout oocyte maturation and preimplantation embryonic development. Beyond their conventional roles as building blocks for protein synthesis [22], amino acids are critical for maintaining redox homeostasis, supporting mitochondrial function, and providing substrates for epigenetic and biosynthetic pathways.

Consistent with the intercellular shuttling of carbohydrates and lipids, amino acids utilized by the oocyte are largely imported from surrounding cumulus cells, forming a core component of the cumulus-oocyte metabolic axis (Fig. 1). During oogenesis, the abundance of most amino acids rises significantly with meiotic resumption and then decreases in fully mature oocytes. While amino acids such as glycine and L-valine continue to increase from the GV to MII stages, others—including 2-aminobutyric acid, cystine, and taurine—show a notable decline [10]. This dynamic remodeling of amino acid pools reflects both changing biosynthetic demands and regulatory roles in cell cycle progression.

A key emerging pathway in oocytes is the serine–glycine–one-carbon (SGOC) metabolism, composed of the folate and methionine cycles. This network provides critical precursors for the synthesis of nucleotides, lipids, and proteins, and generates S-adenosylmethionine (SAM) as a universal methyl donor for DNA and histone methylation [26, 27]. The SGOC pathway is activated during oocyte maturation, as evidenced by increased expression of enzymes involved in both cycles, and likely contributes to epigenetic priming and transcriptional regulation during the maternal-to-zygotic transition (MZT) [10].

Polyamine metabolism, particularly involving spermidine, also exerts important influences on oocyte quality. Spermidine is synthesized from putrescine by spermidine synthase (SRM), and later converted to spermine. It is involved in diverse cellular functions including autophagy, proteostasis, oxidative stress responses, and mitochondrial integrity [28]. Notably, spermidine levels decline with age in the ovary, while supplementation in aged mice improves follicular development, meiotic maturation, mitochondrial structure, and subsequent embryonic development [29]. These effects are attributed to enhanced autophagic flux and mitochondrial quality control [29]. Therefore, amino acid metabolism during oogenesis operates as a tightly regulated system not only to support anabolic growth but also to modulate redox status, epigenetic reprogramming, and organellar function—all of which are critical for ensuring developmental competence.

In contrast to the well-characterized roles of glucose and lipid metabolism, amino acid metabolism in preimplantation embryos has only recently begun to be elucidated in detail. Earlier studies, limited by lower resolution techniques, suggested that amino acid utilization remained relatively static during early cleavage stages [30]. For example, glutamine was thought to be maintained at basal levels throughout development, and the methionine cycle appeared underdeveloped before the blastocyst stage [30].

However, with the advent of high-resolution metabolomics, a more nuanced picture has emerged. Glutamine metabolism is now recognized to be highly stage-specific. At the blastocyst stage, glutamine catabolism is markedly enhanced [5, 31], supported by upregulation of glutamate dehydrogenase 1 (*Glud1*) [5], which drives the conversion of glutamate to α-KG, feeding into the TCA cycle. This metabolic reprogramming supports the energetic and biosynthetic demands of blastocyst formation and lineage segregation.

Together, these findings support the concept that amino acid metabolism is not merely auxiliary but deeply integrated into the regulatory logic of early development. From supporting redox homeostasis and epigenetic remodeling in oocytes to fueling mitochondrial energetics and morphogenesis in embryos, amino acids orchestrate a diverse range of functions essential for reproductive success.

## Mitochondrial remodeling and the metabolism–epigenetics axis across oocyte maturation and embryo development

Mitochondria serve as central metabolic hubs in mammalian oocytes and early embryos, coordinating nutrient catabolism, redox homeostasis, and biosynthetic processes [32]. Beyond their well-characterized roles in ATP generation, mitochondria actively shape the cellular and epigenetic landscape required for successful maturation and developmental progression (Fig. 1). This constitutes a “metabolism–epigenetic” command center that precisely orchestrates meiotic progression, ZGA, and cell fate determination.

During oogenesis, mitochondrial function is closely linked to the NAD^+^ metabolic network [33]. NAD^+^, a key coenzyme in redox and biosynthetic reactions, is synthesized through *de novo* and salvage pathways. Its biosynthesis critically depends on enzymes such as indoleamine 2,3-dioxygenase 1 (IDO1) and quinolinate phosphoribosyltransferase, which regulate *de novo* synthesis, and nicotinamide phosphoribosyltransferase (NAMPT), the rate-limiting enzyme in the salvage pathway that converts nicotinamide to NAD^+^ via nicotinamide mononucleotide (NMN) [34]. These enzymes are co-localized with the mitochondria and are required for maintaining mitochondrial membrane potential, proper spindle formation, and meiotic progression. Deficiencies in NAD^+^ synthesis disrupt mitochondrial distribution and function, impairing oocyte quality and fertilization competence. These results have been demonstrated in mice and pigs [35, 36].

In parallel, oocyte mitochondria are tightly regulated to avoid oxidative damage from ROS, a byproduct of the electron transport chain (ETC). While oocytes were previously thought to possess enhanced ROS-scavenging capacity, recent findings indicate that ROS avoidance is achieved primarily through suppression of ROS generation [37]. Incomplete assembly of Complex I impairs the formation of respiratory supercomplexes, attenuating ETC activity and limiting ROS production [37]. This intrinsic downregulation likely protects mitochondrial DNA (mtDNA) from damage during the prolonged arrest of oocytes in meiotic prophase [37, 38].

As development progresses, mitochondria in early embryos undergo dynamic remodeling that reflects not only shifting energetic demands but also emerging roles in developmental regula­tion. Mitochondrial fission and fusion events, mediated by factors such as dynamin-related protein 1 (DRP1), have long been assumed to primarily support metabolic efficiency [39]. However, the loss of *Drp1* in oocytes likely leads to depletion of TCA cycle intermediates such as succinate and fumarate, alongside reduced levels of SAM, a key methyl donor for epigenetic modifications [40]. These metabolic alterations result in defective DNA methylation and a striking loss of H3K27me3 in female germ cells, culminating in widespread epigenetic dysregulation and thereby affecting postimplantation embryonic development [40].

Mitochondria also modulate the NAD^+^/NADH redox state, which serves as a sensitive rheostat for regulating epigenetic and transcriptional programs. In particular, NAD^+^ availability during minor ZGA is essential for controlling histone acetylation [5]. Through sirtuin 1 (SIRT1)-mediated deacetylation of H3K27ac, NAD^+^ prevents overactivation of the early zygotic genome, ensuring transcriptional precision during the 2-cell stage in mice (2–4-cell stage in humans) [5]. This regulation depends on stage-specific upregulation of Nampt, which peaks at the blastocyst stage to sustain NAD^+^ levels [5]. Intriguingly, the NADH/NAD^+^ ratio decreases sharply at the 8-cell stage and rebounds at the blastocyst stage [5], highlighting a potential role for redox dynamics in guiding cell fate decisions [41].

The interaction between the mitochondria and the nucleus extends far beyond energy provision and the simple exchange of metabolites. A striking example is the discovery of a direct link between metabolism and epigenetic regulation during mammalian ZGA. In mouse 2-cell embryos, a subset of enzymatically active mitochondrial proteins—including PDH and several first-half TCA cycle enzymes—transiently translocate into the nucleus; intriguingly, this relocation is strictly dependent on exogenous pyruvate supply [3]. Within the nuclear compartment, these enzymes catalyze the conversion of pyruvate into acetyl-CoA and α-KG—the essential substrates for histone acetylation and demethylation—thereby initiating ZGA as a pivotal developmental event [3]. This mechanism exemplifies the concept of a “metabolism–epigenetics coupling network,” wherein cells directly regulate chromatin states and transcriptional programs by spatial redeployment of metabolic enzymes to the nucleus, rather than merely altering global metabolite concentrations. Remarkably, a conserved pattern of PDH nuclear localization is also observed in human embryos—occurring specifically at the 4- to 8-cell stage, which coincides with human embryonic genome activation—underscoring the universality and fundamental importance of this regulatory strategy in early development [3].

Together, these findings underscore the emergence of a mitochondria-centered “metabolism–epigenetics coupling network” that orchestrates developmental transitions across the oocyte-to-embryo transition (Fig. 1). Mitochondrial metabolism not only supports energy production but also regulates chromatin remodeling through metabolite availability and redox control. Understanding this coupling provides a conceptual framework for deciphering how metabolic state informs developmental potential, with implications for both fertility preservation and therapeutic intervention in reproductive disorders.

## Metabolism-related diseases

Maternal metabolic disorder status profoundly influences offspring reproductive health. Here, we focus on metabolism-related disorders such as obesity, PMOS, and ovarian aging: maternal obesity is considered an adverse environmental exposure that compromises oocyte quality [42–44]; ovarian aging is closely linked to the progressive decline and eventual depletion of oocyte metabolic reserves [45]; and PMOS directly impairs the oocyte energy supply and metabolic homeostasis through mitochondrial dysfunction [46].

### Obesity

Obesity, as a maternal condition, exerts a significant detrimental impact on oocyte quality and embryonic development. Numerous studies have demonstrated that obesity compromises oocyte health by impairing mitochondrial function and exacerbating oxidative stress [47], resulting in an increased risk of abnormal embryonic development, reduced fertility, and adverse pregnancy outcomes among obese women [42–44]. Single-cell RNA sequencing of female mice with high-fat diet (HFD)-induced obesity revealed downregulation of genes involved in fatty acid metabolism and glycolysis in 8-cell stage embryos, suggesting potential defects in embryonic development and metabolic dysregulation [48]. Meanwhile, in mice with HFD-induced obesity, genes involved in oxidative phosphorylation and glutathione metabolism are not activated, whereas genes associated with DNA damage and repair, autophagy, cellular stress responses, and ferroptosis are upregulated [48]. Conversely, genes related to embryonic development and metabolic pathways are specifically downregulated, indicating that obesity disrupts key events in ZGA and MZT [48].

Moreover, obesity has been shown to affect the epigenetic landscape of both oocytes and embryos. It may increase the expression of DNA methyltransferases (DNMTs), leading to global DNA hypermethylation in oocytes, and such alterations can be partially transmitted to female F2 offspring [49]. During preimplantation embryonic development, obesity changes DNA methylation in early embryos by disrupting α-KG [50]. In addition, NAD^+^ metabolism is also perturbed in obese mice. *Nampt* is significantly downregulated in oocytes from obese mice, resulting in decreased levels of NAD^+^ [51]. This not only leads to a sharp increase in ROS levels but also disrupts metabolic homeostasis in oocytes, ultimately affecting meiotic progression and mitochondrial function, as evidenced by a high incidence of spindle assembly defects and chromosomal misalignment. Notably, ectopic expression of *Nampt* can effectively restore NAD^+^ levels, reduce excessive ROS production, and improve oocyte quality [51].

**Figure 3:**
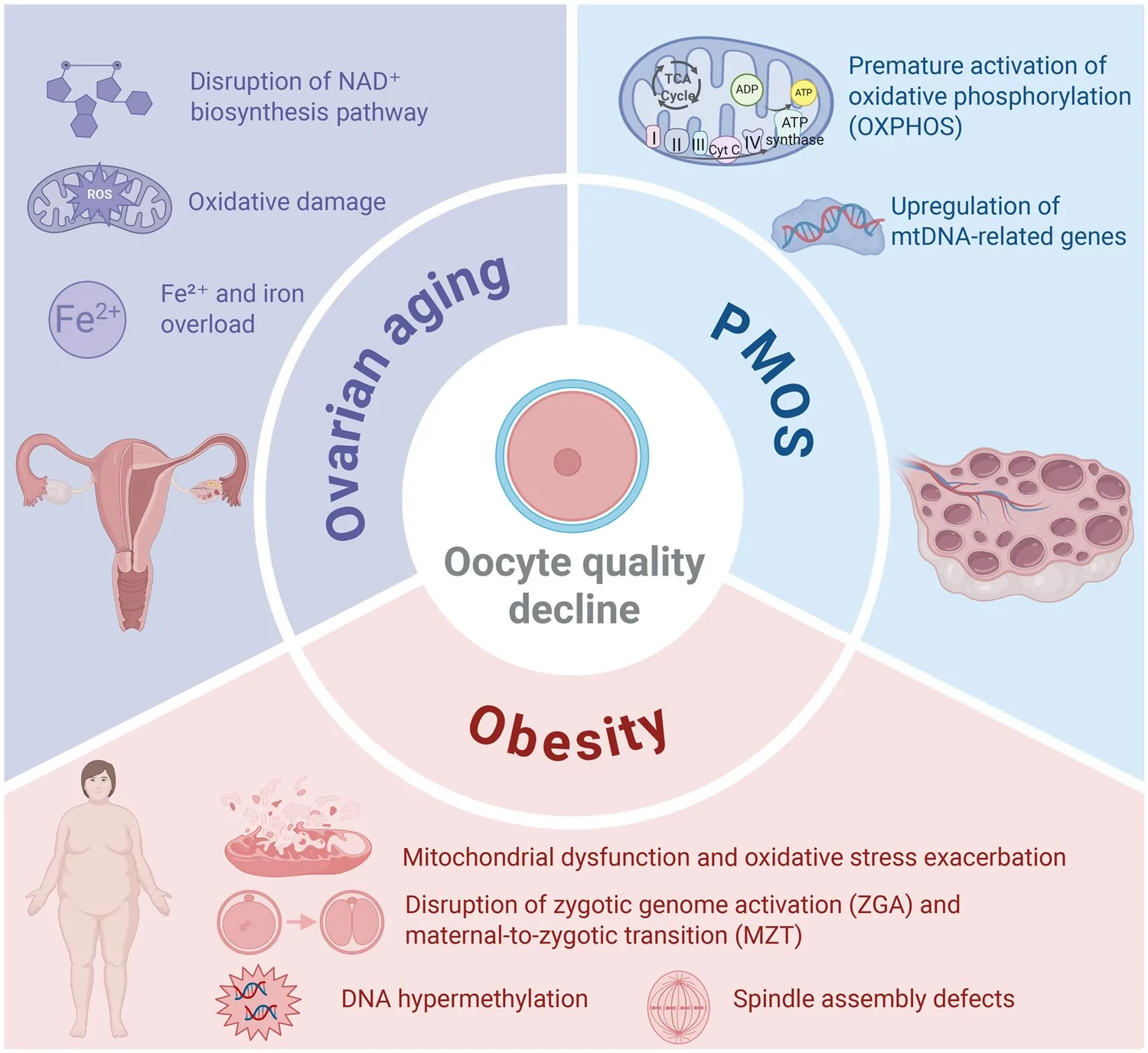
Maternal factors contributing to oocyte quality decline. Schematic summary shows how obesity, ovarian aging, and polyendocrine metabolic ovarian syndrome (PMOS) negatively impact oocyte quality. Created with BioRender.com.

Overall, these findings indicate that obesity can compromise oocyte and embryo quality by impairing mitochondrial function, disrupting metabolic–epigenetic networks (e.g., NAD^+^ depletion and aberrant DNA hypermethylation), and interfering with the processes of MZT and ZGA (Fig. 3).

### Ovarian metabolic failure

In the female reproductive system, the ovary plays a pivotal role. Ovarian metabolic failure denotes a pathological state of compromised bioenergetic adaptability in ovarian tissue, characterized by the collapse of metabolic hubs governing oocyte qua­lity and subsequent impairment of gametogenesis. Clinically, this state manifests as two distinct yet mechanistically convergent disorders: ovarian aging and PMOS. Of note, the ovary is also the first organ to exhibit age-related functional decline, highlighting its unique susceptibility to metabolic deterioration. Numerous studies have demonstrated that ovarian aging leads to reduced oocyte quality. Single-cell transcriptomic profiling of ovarian aging in both monkeys and mice has revealed that oxidative damage is a key factor contributing to age-associated decline in ovarian function [52–54]. In monkeys, downregulation of antioxidant genes is a distinct early hallmark of oocyte aging, resulting in progressive oxidative damage and a decrease in oocyte quantity during ovarian aging [52]. Notably, in advanced maternal age, oxidative damage and impaired mitochondrial function are already evident in aged oocytes at the primordial follicle stage, compelling them to rely on alternative energy sources such as glycolysis and the adenosine salvage pathway [45].

Oocytes and embryos from patients or mouse models with ova­rian aging-related disorders exhibit multiple metabolic abnormali­ties. In terms of iron metabolism, dysregulation in aged mouse oocytes leads to iron overload and excessive accumulation of labile ferrous iron, which triggers oxidative stress and degenerative changes [55]. This cascade results in increased lipid peroxidation, mitochondrial dysfunction, and enhanced lysosomal activity [55]. Regarding NAD^+^ metabolism, ingenuity pathway analysis has shown that all cellular pathways involved in NAD^+^ biosynthesis are disrupted in aged oocytes, leading to a reduction in NAD^+^ levels [33]. Knockout of *Ido1* or *Qprt*, key genes in the *de novo* NAD^+^ biosynthesis pathway, accelerates aging, resulting in decreased ovarian NAD^+^ levels in mice, ultimately leading to mitochondrial dysfunction, accumulation of ROS, and reductions in oocyte qua­lity and quantity [35]. In summary, aged oocytes exhibit metabolic dysregulation characterized primarily by iron accumulation and impaired NAD^+^ biosynthesis (Fig. 3), which directly induces mitochondrial damage and oxidative stress, ultimately resulting in reduced gamete quality.

PMOS is also the most prevalent clinical manifestation of ovarian metabolic failure among women of reproductive age. Patients with PMOS often exhibit impaired oocyte maturation and reduced oocyte quality, resulting in significantly compromised fertility. Single-cell transcriptomic analyses have revealed substantial differences between GV-stage oocytes from healthy individuals and those from PMOS patients [46]. Notably, mitochondrial function—particularly oxidative phosphorylation—appears to be prematurely activated in GV-stage PMOS oocytes [46]. Consequently, mitochondrial dysfunction directly affects oocyte energy supply and metabolic homeostasis, ultimately contributing to reduced oocyte quality [46] (Fig. 3). This finding offers important insights into the mechanisms by which PMOS impairs oocyte competence.

## Promising interventions by altering metabolism

**Figure 4:**
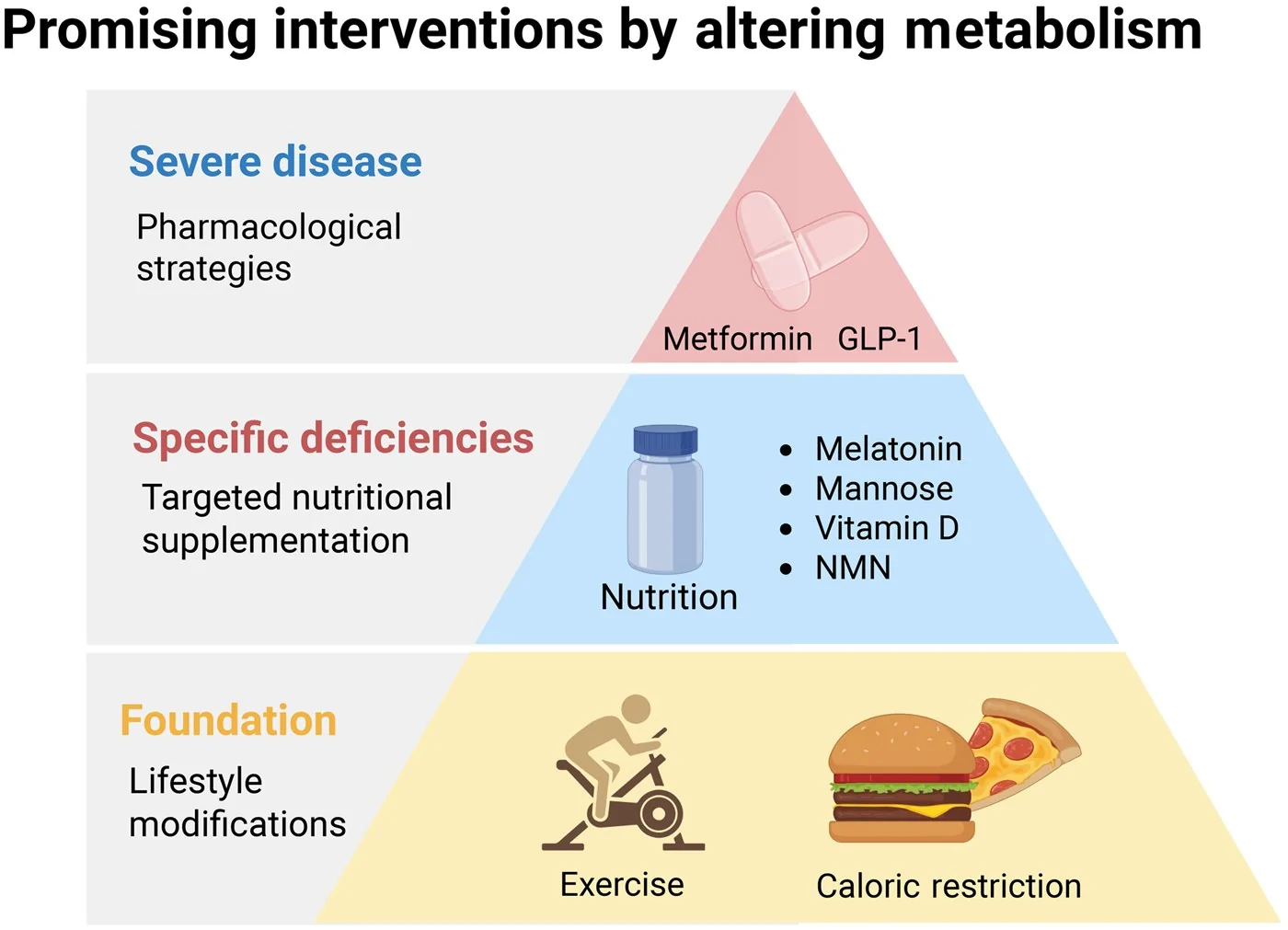
A conceptual framework for hierarchical metabolic interventions in oocyte and embryonic development. Schematic representation of a three-tiered therapeutic pyramid progressing from lifestyle modifications to precise pharmacological targeting is shown. Created with BioRender.com.

Synthesizing the mechanistic insights above, we propose a hierarchical intervention strategy tailored to reproductive metabolic disorders: Tier 1 employs non-invasive lifestyle modifications (e.g., caloric restriction [CR] and exercise) to correct broad metabolic disturbances; Tier 2 utilizes targeted nutritional supplementation (e.g., melatonin [MLT], mannose, vitamin D, and NMN) to address persistent specific deficiencies; Tier 3 escalates to pharmacolo­gical agents (e.g., metformin and glucagon-like peptide-1 [GLP-1] agonists) for precise pathway regulation in severe disease (Fig. 4). This tiered framework embodies a progressive logic, shifting from systemic modulation to precise targeting. Notably, this framework is presented as a conceptual, evidence-informed model rather than a clinical guideline.

### Lifestyle modifications

Anchored at the foundation of our intervention pyramid (Fig. 4), lifestyle modification serves as the first-line, non-invasive stra­tegy to optimize the metabolic microenvironment of oocytes and embryos. CR and exercise interventions lie at the core of this tier, jointly restoring systemic metabolic homeostasis and improving reproductive performance.

Exercise exerts beneficial effects on oocyte quality and embryonic developmental potential by improving systemic metabolic status and modulating the reproductive microenvironment. Regarding the quality of mouse oocytes, exercise significantly enhances both the activity and transcriptional levels of the fatty acid β-oxidation enzyme hydroxyacyl-CoA-dehydrogenase (HADHA) in GV-stage oocytes from HFD-fed mice, thereby promoting lipid metabolism [56]. Additionally, exercise partially restores mitochondrial function by reversing lipid accumulation and reducing ultrastructural abnormalities in mitochondria, such as a decrease in oval-shaped, dumbbell-shaped, and petal-like mitochondria [56]. In terms of embryonic quality, a randomized controlled trial has shown that maternal aerobic, resistance, or combined exercise regimens can influence infant lipid metabolism and mitochondrial dynamics. This is achieved by decreasing mitochondrial coupling and uncoupling respiration capacity in mesenchymal stem cells, thereby improving FAO efficiency and mitigating the adverse effects of a high-fat environment on embryonic development, ultimately enhancing embryo quality [57]. However, some studies have noted that while exercise may alleviate lipotoxic damage to mouse oocytes by optimizing FAO pathways, it has limited efficacy in correcting spindle apparatus abnormalities [56], highlighting the need for complementary and targeted intervention strategies.

Dietary intervention represents another critical strategy, encompassing dietary normalization (DN) and CR. Preconception DN improves insulin sensitivity, glucose tolerance, and serum cholesterol levels, thereby modulating glucose and lipid metabolism [58]. It also improves mitochondrial ultrastructure and reduces intracellular ROS, which together enhance the quality of mouse oocytes and embryos [58]. Intermittent fasting has been shown to significantly improve oocyte and embryo quality by restoring mitochondrial function and reprogramming metabolic pathways [59]. It has been demonstrated in mice that traditional CR, which typically involves a 30%–40% reduction in caloric intake, can improve metabolic parameters but may also pose potential risks to reproductive function [56]. In contrast, studies in porcine models demonstrate that CR enhances cholesterol biosynthesis and steroid hormone metabolism, and alters FAO and steroidogenesis, thereby contributing to improved embryo quality [60]. With respect to glucose metabolism, CR significantly improves glucose tolerance and insulin sensitivity, which in turn ameliorates offspring phenotypes in mice [61]. Moreover, it improves mouse oocyte quality primarily by reducing ROS and partially restoring mitochondrial activity, but fails to reduce LD volume [62], reflecting the inherent limitations of CR-based strategies. These experimental findings are inconsistent; therefore, in the context of improving the metabolic environment of oocytes and embryos, whether to adopt a normal diet or CR remains to be further investigated.

Despite significant progress in recent years regarding the roles of exercise and dietary interventions in regulating oocyte and embryonic metabolism, their specific impact on human oocyte quality is largely inferred from preclinical research. Similarly, structured exercise regimens improve systemic insulin sensitivity, yet direct evidence linking exercise protocols to enhanced embryogenesis in humans remains scarce. The current body of evidence remains limited. Thus, although these interventions serve as fundamental strategies, they require careful evaluation under distinct clinical scenarios. Future research should also prioritize combined lifestyle interventions to potentially synergistically enhance oocyte and embryo quality.

### Nutrients

When foundational Tier 1 lifestyle modifications fail to resolve specific molecular deficits, targeted nutritional supplementation represents a proposed second-tier, mechanism-based approach within our conceptual intervention framework (Fig. 4). This stra­tegy employs specific bioactive compounds—including MLT, mannose, vitamin D, and NMN—to alleviate oxidative stress, optimize mitochondrial respiration, and drive cytoplasmic and nuclear maturation, primarily in preclinical models. By replenishing these critical substrates, nutritional modulators offer a refined approach to rectifying specific metabolic deficits in oocytes and embryos. Notably, these nutritional supplements have demonstrated therapeutic potential primarily in preclinical studies.

Among them, MLT stands out as a critical antioxidant and metabolic regulator involved in buffalo oocyte developmental regulation [63]. During the gonadotropin-dependent phase, follicle-stimulating hormone induces a rise in MLT levels in ova­rian homogenates. Short-term exogenous MLT supplementation in murine models significantly increases cytoplasmic nutrient content, including total protein and triglycerides, leading to enlarged oocyte volume and enhanced mitochondrial energy metabolism efficiency [64]. Concurrently, it reduces ROS levels and markedly elevates mtDNA copy number, membrane potential, and ATP production [64]. In contrast, MLT-deficient mice exhibit abnormal phenotypes such as reduced oocyte volume and impaired mitochondrial function, all of which can be reversed by exogenous MLT supplementation [64]. Salidroside, the major active component of *Rhodiola rosea* with low toxicity and few side effects, has long been regarded as an effective remedy for altitude sickness [65]. Recent evidence suggests that salidroside also plays a criti­cal role in regulating oocyte development by enhancing mitophagy. During aging, impaired mitophagy in oocytes leads to mitochondrial dysfunction; salidroside restores mitochondrial homeostasis, reduces ROS levels, and significantly improves oocyte developmental competence [65]. During embryonic deve­lopment, mannose has also been identified as a critical nutrient factor in regulating mesodermal fate. Studies using mouse embryonic stem cell models have demonstrated that mannose supplementation can rescue mesodermal differentiation defects induced by 2-DG, restoring *T/Bra* expression and axial elongation [66]. This finding not only highlights a signaling role for mannose metabolism in cell fate determination but also provides a theoretical basis for supplementing mannose or other glycosylation precursors in human embryo culture to optimize developmental outcomes.

Supplementation with key nutrients such as folic acid and vitamin D also holds particular significance. In protein-restricted murine models, folic acid supplementation reverses serine meta­bolic abnormalities in oocytes, restores the methylation rate of homocysteine to normal levels, and ameliorates mitochondrial cristae structural defects [67]. Vitamin D, by activating the vitamin D receptor, upregulates antioxidant enzyme expression (e.g., superoxide dismutase 2 [SOD2]) in goat oocytes [68]. In vitamin D-deficient rat models, supplementation reduces oocyte ROS levels and increases blastocyst formation rates [67].

Furthermore, prior studies have indicated that supplementation with the NAD^+^ precursor nicotinic acid significantly enhances the developmental competence of oocytes and early embryos in obese mice [51]. Direct supplementation with NMN has also been shown to boost mitochondrial function, improve energy metabolism, reduce inflammation, and ultimately improve oocyte quality and ovarian health in aged mice [4]. These findings highlight the therapeutic potential of targeting NAD^+^ metabolism to ameliorate obesity-related reproductive dysfunction.

Collectively, these nutritional interventions modulate oocyte and embryonic metabolism via distinct mechanistic pathways. Although preclinical findings provide a conceptual basis for improving reproductive health, robust clinical evidence in humans remains limited. Future studies must prioritize rigorous preclinical validation and early-phase clinical trials to elucidate their mechanisms of action and optimize intervention strategies prior to any broader clinical application.

### Pharmacological intervention

Occupying the apex of our intervention pyramid, pharmacological strategies represent the third-tier, high-efficacy approach but necessitate stringent evaluation. This tier is reserved for cases where foundational lifestyle modifications and targeted nutritional supplementation prove insufficient to reverse severe reproductive metabolic disorders. This tier centers on two clinically investigated agents—metformin and GLP-1 receptor agonists. These agents halt disease progression by restoring mitochondrial homeostasis, correcting insulin resistance, and normalizing follicular metabolic microenvironments.

Among these, the mechanism of metformin action has been extensively validated. In a dehydroepiandrosterone-induced PMOS mouse model, metformin significantly reduces oxidative stress levels and modulates epigenetic alterations, thereby restoring the developmental competence of oocytes affected by PMOS [69]. Furthermore, in oocytes from aged mice, metformin improves mitochondrial function by reducing mitochondrial ROS via regulation of SOD2^K68^ acetylation by SIRT3, effectively decreasing apoptosis, increasing autophagy, and ultimately improving oocyte quality in aged animals [70]. However, precise control of drug dosage is critical. *In vitro* culture of murine 2-cell embryos exposed to 500 μg/mL metformin induces complete developmental arrest at the compaction stage, underscoring the need for meticulous dose selection in clinical applications to avoid detrimental effects on early embryonic development [71]. In addition to metformin, several other agents with similar or even superior effects have been identified. For instance, in PMOS patients undergoing intracytoplasmic sperm injection, sitagliptin demonstrated greater efficacy than metformin in improving oocyte maturation and embryo quality [72]. Similarly, pachymic acid has been shown to enhance the endocrine environment and oocyte quality in PMOS mice, particularly by alleviating adipose tissue inflammation and mitochondrial dysfunction—areas in which it outperforms metformin [73]. Moreover, in a randomized controlled pilot study involving PMOS patients, sitaformin has exhibited greater effectiveness than metformin in reducing the number of immature oocytes and improving embryo quality [74].

In recent years, GLP-1 receptor agonists have attracted increasing attention in the treatment of PMOS and obesity-related reproductive disorders due to their unique regulatory effects on glucose and lipid metabolism. Animal studies have shown that preconception treatment of obese female mice with semaglutide alleviates lipid and glucose metabolic disturbances, enhances insulin sensitivity and glucose tolerance, and reduces excessive ROS levels in oocytes prior to conception. This, in turn, leads to improvements in adipose tissue inflammation, insulin resistance, and metabolic dysfunction in offspring [75]. However, the underlying mechanisms by which GLP-1-based therapies influence oocyte and embryonic development remain largely unexplored, necessitating further high-quality basic and clinical research.

### Metabolic regulation in ART

Assisted reproductive technology (ART) is not a metabolism-targeted therapy. However, *in vitro* maturation, *in vitro* fertilization, and embryo culture provide a controlled *in vitro* platform for modifying the metabolic environment of oocytes and embryos. The evolution of ART reflects ongoing advances in understanding the metabolic requirements of oocytes and early embryos. Early studies in mice showed that the substrates used by oocytes and embryos change across preimplantation deve­lopment [76, 77], and later work extended these observations to mammalian and human embryo culture [78, 79].

Pyruvate and lactate are important oxidative substrates in oocyte maturation and early cleavage development, while glucose utilization generally increases during compaction and blastocyst formation [77, 80, 81]. Amino acids additionally contribute to biosynthesis, osmoregulation, and redox homeostasis [79]. These findings help shape embryo culture media by showing that both nutrient composition and the timing of exposure matter. Sequential media adjust the culture environment as embryos develop, whereas single-step media provide multiple substrates throughout the culture period. Both approaches are now used clinically, although the actual concentrations of nutrients vary considerably among commercial media [82, 83]. Studies of culture conditions now examine not only cleavage and blastocyst formation but also mitochondrial function, oxidative stress, meta­bolic homeostasis, and possible epigenetic effects [83, 84].

More recent studies have tested whether oxygen tension, substrate concentration, exposure time, and metabolic supplements can further improve oocyte and embryo development. MLT is one of the few supplements tested in human embryo culture, although only in a small number of studies [85]. Most evidence for L-carnitine still comes from non-human mammalian oocytes and embryos [86], and NAD^+^ precursors have mainly been studied in aged mice [87, 88]. These approaches may be particularly relevant for women with obesity, PMOS, or advanced reproductive age, because their oocytes may already show mitochondrial and metabolic abnormalities before oocyte retrieval [45, 46, 51]. However, disease-specific culture strategies nowadays are still poorly developed, and it is unclear whether short-term manipulation of the culture environment can reverse defects acquired during follicular growth. Many studies have focused on MII rates, ROS levels, ATP content, or blastocyst formation, whereas evidence for improved clinical pregnancy, live birth, and long-term offspring health remains limited [63, 70].

## Conclusions, unresolved questions, and future directions

Metabolic regulation from oocyte maturation to early embryogenesis is critical for ensuring reproductive success and deve­lopmental competence. This review underscores the dynamic interplay of glucose, lipid, and amino acid metabolism and the central role of mitochondria in orchestrating ATP production, redox balance, and epigenetic remodeling across developmental stages. During oocyte maturation, metabolic networks are predominantly governed by mitochondrial TCA cycle activity and the PPP, which collectively coordinate ATP production, redox homeostasis, and epigenetic modifications to establish developmental competence. In contrast, early embryogenesis features a stage-specific metabolic transition—such as the activation of glycolysis and remodeling of lipid metabolism at the blastocyst stage—which drives cell fate determination and morphogenesis via a metabolism–epigenetics interaction network. The interplay among these metabolic pathways not only ensures sufficient energy supply and biosynthetic precursors but also precisely modulates epigenetic modifications and redox balance, ultimately influencing developmental potential.

Increasing evidence supports a causal link between meta­bolic dysregulation and reproductive disorders. Obesity, for instance, exacerbates oxidative stress through disrupted NAD^+^ metabolism, resulting in meiotic defects in oocytes. Similarly, ovarian aging is accompanied by mitochondrial dysfunction and ferroptosis, which together promote primordial follicle depletion. These findings highlight promising therapeutic targets—including NAD^+^ precursor supplementation, spermidine-mediated autophagy enhancement, and iron chelation—all of which have shown potential in animal models to restore oocyte and embryo quality. Key questions remain, however. Can such targeted metabolic interventions (e.g., NAD^+^ precursors, one-carbon cycle metabolites, and lipid modulators) reliably improve the developmental competence of human oocytes and embryos? From a basic research perspective, how do oocytes and embryos sense and adapt to dynamic fluctuations in their microenvironment, including variations in follicular fluid composition and maternal systemic factors? It is plausible that oocytes and embryos have evolved robust strategies to tolerate or buffer against these changes—understanding these adaptive mechanisms will be critical for designing effective, metabolism-based therapies.

Furthermore, current studies remain heavily reliant on mammalian models, such as mice, and the limited availability of human oocytes and embryos constrains cross-species validation and clinical translation. Critical questions remain about species-specific differences in metabolism. How does human oocyte and embryo metabolism diverge from that of commonly studied mouse models? Can we implement metabolomic profiling across more developmental stages of human oocytes and embryos? Advances in low-input and single-cell technologies, including newly introduced single-embryo proteomics [89] and the concomitant proteomic perspective on maternal‑to‑zygotic proteome dynamics [6], illustrate how the integration of multi-omics datasets promi­ses to address these gaps. These mechanistic insights may guide the optimization of oocyte maturation and embryo culture in ART, although the safety and clinical efficacy of metabolism-based strategies require rigorous validation. In conclusion, a deeper understanding of the metabolic regulation underlying oocyte maturation and embryonic development not only sheds light on maternal and fetal energy demands during reproductive processes but also provides a critical theoretical foundation for improving reproductive outcomes and addressing infertility.
